# Intelligence, Correspondence, &c.

**Published:** 1836-10-01

**Authors:** 


### Intelligence, Correspondence, &c.

Dr. SIGMOND's Letter to Dr. GOLDING came to hand.——Dr. THACKER's Communications came far too late, the last sheet of the Journal being closed.

Several Works arrived since the Bibliographical Record was put to press.

LITERARY INTELLIGENCE.

The Report of Sir David Barry and Dr. Corrie on the Medical Charities of Ireland will shortly be published. These Gentlemen were appointed by Government Commissioners for investigating the Management of Hospitals and Asylums.

A Bed-side Manual of Physical Diagnosis, by Charles Cowan, M.D. It is in the press, and will very shortly be published.

